# Three waves of the COVID-19 pandemic

**DOI:** 10.1136/postgradmedj-2020-138564

**Published:** 2020-08-18

**Authors:** Temitope Fisayo, Sonia Tsukagoshi

**Affiliations:** King’s College London School of Medicine, London, UK; Wonca Europe, London, UK

COVID-19 was first reported in the UK at the end of January 2020 and lockdown announced on 23 March 2020. Many of us have uttered the words ‘when this is over’, but what does that really mean? As the first-, second- and third-order impacts of the virus manifest over different time frames, this pandemic will not necessarily be ‘over’ until we are through the impact of the ‘third wave’ of the COVID-19 pandemic.

We are currently experiencing the effects of the first wave, where deaths and disability are directly linked to COVID-19. Alongside the atrocious death toll, an as yet untold number of people are living with the lasting aftermath of a severe acute respiratory syndrome coronavirus 2 infection—for some, even mild COVID-19 can be debilitating for months on end, even after they are clinically cured of the infection.^1^

The second wave refers to those who will suffer in the medium-term due to measures taken to limit the spread of COVID-19. It includes, among many others, those who delay presenting to healthcare facilities for fear of COVID-19 infection; those with progressive diseases whose appointments are rescheduled; and those who miss routine screening. The question of how doctors, particularly those working in primary care, will navigate the backlog remains unanswered.

The third wave is the effect of virus on the social determinants of health, and its effects on the next generation.^2^ The virus will worsen health inequalities through severe economic injury.^3^ It is the sectors that rely on low-paid staff (often women, young people and Black, Asian and minority ethnic (BAME) people) that will take longest to recover from the predicted deep economic recession.^3^ The health impacts caused by this worsening of economic conditions will be complex, but it is likely that groups that are at the intersection between poverty and poor health that will suffer most.^3^

Children have already been locked out of schools. Despite teachers’ best efforts, there will be children who will never compensate for their months of lost education, who will be locked out of well-paid jobs and locked into a lifetime of poorer health as a result.^4^ Early reports suggest privately educated children have been able to continue learning from home, whereas their state-schooled counterparts are missing out.^5^ Of course, it is a lot easier to transition to online learning if you have your own laptop and your parents are able to keep an eye on your activities while they work from home.

Parental loss of income will impact on children’s diets, housing and educational opportunities. Possible resultant cuts to public funding^6^ could lead to youth centres being shuttered; libraries never reopening; and the end of council-funded arts programmes (if they existed earlier). Children would lose even more spaces to socialise safely, potentially having to settle for obesogenic spaces such as fried chicken shops.^7^

For vulnerable children living in temporary accommodation, losing access to routine check-ins from health services, legal advice and immigration support could have consequences that mark them for life.^8^ Lockdown means an increased risk of child abuse and domestic violence, both adverse childhood experiences with demonstrated repercussions in later life.^6^ Changes in law aimed at accommodating social distancing have also weakened statutory protections for some children: it is as yet unclear when these changes will be refined or reversed.^9^

Even beginning to trace the lasting impacts of the COVID-19 pandemic can be overwhelming. Yet this is the meaning of a pandemic: the virus has got into everything. It’s in our funding decisions, our legal protections, our hearts our minds. The pandemic cannot be ‘over’ until it is reckoned within every sphere of our lives.

But we mustn’t lose sight of that crucial thing about the third wave—it’s still far out at sea. The future is not foregone. We can act now, to mitigate economic injury, to prioritise the return of libraries, youth and community centres. The third wave doesn’t have to crash on undefended shores.
